# Anesthesia management in a patient with very long-chain acyl-Coenzyme A dehydrogenase deficiency

**DOI:** 10.1186/s40981-020-00379-8

**Published:** 2020-09-16

**Authors:** Haruyuki Yuasa, Yukio Onoda, Atsuhiro Kitaura, Takashi Mino, Shota Tsukimoto, Shinichi Nakao

**Affiliations:** 1 Department of Anesthesiology, Faculty of Medicine, Kindai University, 377-2 Ohonohigashi, Osakasayama, Osaka, 589-8511 Japan; 2 Onoda Clinic, 1917-1 Yuasa Yuasa-cho Arita-gun, Wakayama, 643-0004 Japan

**Keywords:** Very long-chain acyl-Coenzyme A dehydrogenase deficiency, Rhabdomyolysis, Glucose, Lactate

## Abstract

**Background:**

In a patient with very long-chain acyl-Coenzyme A dehydrogenase (VLCAD) deficiency, metabolism of fatty acids is impaired and a supply of alternative energy is limited when glucose level is insufficient on starvation.

**Case presentation:**

A 37-year-old woman with VLCAD deficiency was diagnosed with an ovarian cyst and was scheduled for laparoscopic ovarian cystectomy. Glucose was administered intravenously with the start of fasting. Anesthesia was induced with remifentanil, midazolam, and thiamylal, maintained with desflurane and remifentanil. Body temperature was maintained at 36.2–36.7 °C. During anesthesia, hypoglycemia did not occur, creatine kinase levels were in the normal range, and myoglobinuria was not detected. No shivering was observed after extubation.

**Conclusions:**

Glucose was administered to avoid perioperative hypoglycemia. Body temperature was controlled to avoid shivering, which would otherwise increase skeletal muscle energy needs. Blood creatine kinase did not increase, and myoglobinuria was not detected; thus, rhabdomyolysis was unlikely to develop.

## Background

Very long-chain acyl-Coenzyme A dehydrogenase (VLCAD) deficiency is one of the disorders of mitochondrial fatty acid metabolism [1]. β-Oxidation of fatty acids is impaired, and alternative energy supply would be insufficient on starvation in affected patients. In particular, since skeletal muscle mainly depends on fatty acid β-oxidation for energy supply for its contraction, rhabdomyolysis may occur when energy deficiency becomes remarkable [2]. We describe anesthesia in a patient with VLCAD deficiency, where rhabdomyolysis was prevented by continuous glucose administration and body temperature control.

## Case presentation

A 37-year-old woman (height, 158 cm; body weight, 50 kg; ASA physical status class II; no systemic complications other than VLCAD exist) with VLCAD deficiency was diagnosed with an ovarian cyst and was scheduled for laparoscopic ovarian cystectomy. Rhabdomyolysis due to VLCAD deficiency first appeared at age 6 with skeletal muscle pain and myoglobinuria after swimming. Since then, she has exhibited similar symptoms several times a year during fasting, exercise, and mental stress. At the age of 31, VLCAD deficiency was diagnosed by acylcarnitine profile analysis. At the same time, genetic mutations c.1349G>A (p.R450H) and c.1639G>A (p.V547M) were identified in her ACADVL gene.

Glucose was administered at 2 mg^−1^ kg^−1^ h^−1^ intravenously to prevent hypoglycemia with the start of fasting at 9 p.m. on the night before the surgery. On the day of surgery, when the patient entered the operating room, her blood glucose level was 108 mg dL^−1^  (Table 1). Glucose administration was increased to 4 mg^−1^ kg^−1^ h^−1^. Anesthesia was induced with 0.5 μg^−1^ kg^−1^ min^−1^ of remifentanil, 3 mg of midazolam, and 200 mg of thiamylal, and muscle relaxation was achieved by 30 mg of rocuronium. After intubation with a cuffed tracheal tube, mechanical ventilation was started, and anesthesia was maintained with 40% oxygen and 5% desflurane in combination with the continuous infusion of 0.25–0.3 μg^−1^ kg^−1^ min^−1^ of remifentanil. Routine monitoring of vital signs and invasive radial artery pressure was performed. Blood glucose, creatine kinase, myoglobinuria, and lactate were monitored during anesthesia (Table 1). To avoid postoperative shivering, body temperature was maintained at 36.2–36.7 °C (Table 1). Acetaminophen (1000 mg) and buprenorphine (0.1 mg) were administered for postoperative analgesia. At the end of the surgery, administration of desflurane and remifentanil was stopped, and rocuronium was antagonized by 200 mg of sugammadex. Surgery time was 53 min, and anesthesia time was 1 h 59 min. No shivering was observed after extubation. Glucose was administered at 2–4 mg^−1^ kg^−1^ h^−1^ until oral intake was started. The blood glucose level of the patient was 142 mg dL^−1^  about 1 h after the surgery. The patient was discharged 2 days after the surgery.

**Table 1:** Values of parameters during anesthesia

	Before induction	At the start of surgery	30 min after the start of surgery	End of surgery
Glucose (mg/dL)	108	138	190	109
Creatine kinase (U/L)	72	66	65	68
Lactate (mM)	0.9	1.0	1.3	1.6
Myoglobinuria	Not detected	Not detected	Not detected	Not detected
Body temperature (Celsius)	36.7	36.5	36.5	36.2

## Discussion

VLCAD deficiency is one of disorders of fatty acid oxidation in the mitochondria and was first reported in 1993 [1]. This disease is a hereditary disease, which shows autosomal recessive inheritance, and the frequency of occurrence is one in 31,500–85,000 births [3, 4]. VLCAD is a long-chain fatty acid-based enzyme that is localized in the mitochondria and catalyzes the first reaction in fatty acid β-oxidation. Healthy people can degrade fatty acids by β-oxidation to supply energy (ATP) when there is a shortage of energy supply from glucose such as from fasting, intense exercise, and infection [5]. However, in a patient with VLCAD deficiency, as β-oxidation is impaired, ATP is not normally produced from fatty acids in the liver and muscles [5]. ATP in such patients is mainly obtained by metabolism of glucose through glycolysis, citric acid cycle, and electron transfer system. In addition, gluconeogenesis is impaired in this disease because acetyl-CoA generated by β-oxidation is required for gluconeogenesis. Significant ATP deficiency in muscles results in rhabdomyolysis. Because fatty acids are not decomposed by β-oxidation, ketones are not produced, resulting in hypokalemia, hypoglycemia, and fatty degeneration in any organs due to accumulation of undecomposed fatty acids, which can cause myocardial damage, skeletal muscle weakness, and fatty livers [6]. The degree of residual activity of VLCAD affects the onset of the disease and the severity of symptoms. Patients with little residual activity develop myocardial symptoms (hypertrophic cardiomyopathy) and liver symptoms (hypoketotic hypoglycemia-Reye syndrome) during the neonatal period [7], patients with moderate residual activity mainly develop liver and skeletal muscle symptoms (rhabdomyolysis) after infancy [8], and patients with relatively high residual activity mainly develop skeletal muscle symptoms after puberty [9].

Since the present case developed skeletal muscle symptoms at the age of 6 years, it was considered that the residual activity of VLCAD remains moderate. In addition, a R450H ACADVL gene mutation found in this case is often found in patients with skeletal muscle symptoms [10].

The ultimate goal of anesthetic management in a patient with VLCAD deficiency is to prevent rhabdomyolysis by supplying sufficient glucose and suppressing the increase in energy demand. In order to accomplish this purpose, adequate glucose supply and avoidance of increased energy demand by postoperative shivering are necessary. More stringent management of glucose supply and energy demand is required when residual activity of VLCAD is reduced. In this case, supplementation of intravenous glucose infusion was performed continuously from the start of fasting 6 h before the surgery to the start of oral intake. Blood glucose levels ranged from 108 to 190 mg dL^−1^ and did not result in hypoglycemia (Table 1).

Shivering generates heat by involuntary movements of skeletal muscles. If the energy demand of skeletal muscles markedly increases, then rhabdomyolysis may occur in the patients. Therefore, the body surface was actively warmed during anesthesia, and the body temperature was maintained around 36.5 °C (Table 1). Fortunately, we were able to achieve our purpose. As a result, no increase in creatine kinase or expression of myoglobinuria was observed during anesthesia (Table 1) and rhabdomyolysis did not occur.

As for anesthetics, benzodiazepines, barbiturates, non-depolarizing muscle relaxants, and opioids have been previously used without problems [6, 11–13]. Propofol was not selected in the present case because it contains long-chain fatty acids in the solvent [6, 13]. There have been conflicting results of using inhaled anesthetics for patients with VLCAD deficiency. A few papers advised avoidance of inhaled anesthetics for these patients, because some patients with VLCAD deficiency developed rhabdomyolysis during anesthesia with inhaled anesthetics [12, 14]. However, the rhabdomyolysis seemed to be induced not by inhaled anesthetics but by inadequate supply of carbohydrate. Kleemann et al. [15] measured plasma concentration of free fatty acids as a marker of catabolism secondary to stress during enflurane anesthesia in patients without VLCAD deficiency and reported that free fatty acids transiently increased around tracheal intubation, but decreased 10 min following the start of enflurane and intraoperatively, suggesting that enflurane anesthesia could suppress stress-induced catabolism [15]. Almost all papers demonstrated that inhaled anesthetics were safely used for patients with VLCAD deficiency [6, 13, 16].

Iwata et al. [16] reported an intraoperative lactate increase in a patient with VLCAD deficiency. In the present case, plasma lactate level increased to 1.6 mM, the normal upper limit, at the end of the surgery (Table 1). Thus, if the surgery had been longer and much more invasive, it would have exceeded the normal range. When the plasma lactate level exceeds the normal limit (1.5 mM) in patients with VLCADD during surgery, we should suppress catabolism by means of glucose infusion and attenuation of physical stress, such as blood pressure increase, heart rate increase, and body temperature elevation.

In conclusion, we experienced anesthesia management in a patient with VLCAD deficiency. Continuous glucose administration was used to avoid perioperative hypoglycemia. Body temperature was controlled to avoid shivering, which would otherwise increase skeletal muscle energy needs. Creatine kinase level did not increase, myoglobinuria was not detected, and thus, rhabdomyolysis was unlikely to develop.

## Data Availability

Not applicable

## Abbreviations

VLCAD: Very long-chain acyl-Coenzyme A dehydrogenase
